# Sustained Reduction of Seizures in Patients with Intractable Epilepsy after Self-Regulation Training of Slow Cortical Potentials – 10 Years After

**DOI:** 10.3389/fnhum.2014.00604

**Published:** 2014-08-08

**Authors:** Ute Strehl, Sarah M. Birkle, Sonja Wörz, Boris Kotchoubey

**Affiliations:** ^1^Institute of Medical Psychology and Behavioral Neurobiology, University of Tuebingen, Tuebingen, Germany; ^2^LWL-University Clinic of Hamm for Child and Adolescent Psychiatry, Psychotherapy and Psychosomatic Medicine at the Ruhr-University Bochum, Bochum, Germany

**Keywords:** epilepsy, long-term follow-up, neurofeedback, slow cortical potentials, seizure reduction

## Abstract

The aim of this study was to determine whether the reduction of seizures in patients with intractable epilepsy after self-regulation of slow cortical potentials (SCPs) was maintained almost 10 years after the end of treatment. Originally, 41 patients received training with SCP-neurofeedback. A control group of 12 patients received respiratory feedback while another group of 11 patients had their anticonvulsant medications reviewed. Nineteen patients in the experimental group participated at least in parts of the long-term follow-up, but only two patients from each control group agreed to do so. The follow-up participants completed the same seizure diaries as in the original study. Patients of the experimental group also took part in three SCP-training sessions at the follow-up evaluation. Due to the small sample size, the results of participants in the control groups were not considered in the analysis. A significant decrease in seizure frequency was found about 10 years after the end of SCP treatment. The clinical significance of this result is considered medium to high. All patients were still able to self-regulate their SCPs during the feedback condition. This success was achieved without booster sessions. This is the longest follow-up evaluation of the outcome of a psychophysiological treatment in patients with epilepsy ever reported. Reduced seizure frequency may be the result of patients continued ability to self-regulate their SCPs. Given such a long follow-up period, the possible impact of confounding variables should be taken into account. The small number of patients participating in this follow-up evaluation diminishes the ability to make causal inferences. However, the consistency and duration of improvement for patients who received SCP-feedback training suggests that such treatment may be considered as a treatment for patients with intractable epilepsy and as an adjunct to conventional therapies.

## Introduction

About one-third of patients with epilepsy do not respond to anti-epileptic drugs (AED). New AED and surgery are the standard treatment for those patients but still a considerable percentage of patients are left being drug resistant. The majority of these patients suffer from focal seizures. Especially for these kind of seizures feedback of brain activity (EEG-feedback, mostly called neurofeedback) has been developed and evaluated since last decades of the last century independently by two different research groups. A decrease of seizures after enhancement of the sensory motor rhythm in patients with poorly controlled epilepsies was reported in 1972 for the first time (Sterman and Friar, 1972). This treatment was motivated by earlier animal studies. Here, it was shown that cats being trained to enhance their SMR were more resistant to the exposition to a rocket fuel called monomethylhydrazine. Compared to cats that had not undergone SMR-training these cats showed elevated seizure thresholds and longer latencies (Sterman et al., 1969; Sterman, 1976). It was concluded that SMR-training decreased seizure susceptibility. Protocols for increasing SMR-activity and in some studies decreasing slow rhythms (delta and theta) of EEG have been used in the following years for research and practice mainly in the United States (for a review see Tan et al., 2009). In parallel to this development, Rockstroh et al. (1993) were able to reduce seizures after feedback of slow cortical potentials (SCPs).

Slow cortical potentials are very slow phenomena of the EEG with a frequency near 0.01 Hz; their duration is up to several seconds. Negative shifts reflect low excitation thresholds that precede epileptic seizures (Ikeda et al., 1996). As the above mentioned trial by Rockstroh et al. (1993) was not controlled Kotchoubey et al. (2001) wanted to replicate the results in a controlled study. Patients being diagnosed as having focal epilepsies were referred to the study centers (University of Tuebingen, Neurological Clinic Weissenau, and Center for Epilepsies (Epilepsiezentrum Kehl-Kork) by their neurologists or general practitioners. The study centers did the additional screening with regards to the following selection criteria: partial epilepsy being diagnosed at least 2 years ago; no reduction of seizures after 2 years of pharmacological treatment (i.e., the epilepsy was declared as pharmaco-resistant); age between 14 and 50 years; IQ above 80; no change in medication beginning from baseline until 1 year after the end of treatment (SCP and RESP groups); no psychogenic seizures, no psychiatric disease according to DSM IV (American Psychiatric Association, 1994).

To maximize effects of expectancy patients were allowed to choose 1 out of 3 treatments: while 41 patients received feedback of SCPs, 11 were trained to self-regulate breathing rate and amount of exhaled CO_2_ (respiratory feedback – RESP) and another group of 12 patients had their medications reviewed (MED). All groups were provided with the same amount of psychosocial treatment: while the feedback groups (SCP and RESP) were trained to apply the self-regulation skills in real-life situations, the MED group received sessions of art therapy, occupational therapy, physiotherapy, and training of social competence.

It was shown that patients acquired the SCP self-regulation skill, which even improved during the follow-up period (Kotchoubey et al., 1997). A 1 year follow-up showed that seizure frequency decreased significantly in the SCP and MED groups, only (Kotchoubey et al., 2001). Psychological variables (depression, locus of control) improved in all treatment groups (Strehl et al., 2005) but IQ changed in the SCP group only (Strehl et al., 2011). About 9 years after the end of treatment, we wanted to know whether the seizure control was maintained in the SCP group and to compare their results with the results of the other two groups. The study was approved by the local Ethics Committee of the Faculty of Medicine, University of Tuebingen and all patients signed informed consent.

## Materials and Methods

### Patients

All patients that had finished the 12 months follow-up were asked whether they would like to take part. A total of 23 responded. Table 1 gives an overview of patients in the treatment groups.

**Table 1:** **Participants in the original study and the follow-up evaluations**.

Participants	SCP	MED	RESP
Original study	41	12	11
1 year Follow-up	34	11	7
Willing to take part in 8 years FU	19	2	2
Percentage, compared with the beginning of treatment	46	17	18
Percentage, compared with 1 year FU	56	18	29
Completed 8 years FU	16		
Percentage, compared with the beginning of treatment	39		
Percentage, compared with 1 year FU	47		

Due to the small number of RESP and MED patients willing to take part in the follow-up-study only SCP patients were included. As depicted in Table 1, these 19 subjects account for 39% of those patients who joined the study and 47% of those who took part in the 1 year follow-up evaluation. From these 19 subjects, 16 submitted seizure data and took part in the SCP sessions. The characteristics of these participants are described in Table 2.

**Table 2:** **Characteristics of long-term follow-up participants**.

Sex	7 Female; 9 male
Age at long-term follow-up	Mean 46; range 31–59 years
Education	Mean 11 years (SD 2)
First seizure (age)	Birth – 43 years
Seizure type (number of patients)	2 Simple focal
	6 Simple and complex focal
	2 Simple, complex, and secondary generalized
	6 Complex focal
Seizures/week (baseline)	Mean 3.49, range 0.08–25.58
Number of anti-epileptic drugs (AED) (baseline)	Mean 2.32, range 1–4
Number of anti-epileptic drugs (AED) (long-term follow-up)	Mean 2.12, range 1–4
Surgery after 1 year FU (number of patients)	3

### Treatment

As described in detail elsewhere (Kotchoubey et al., 2001) patients received treatment in either of three groups:

MED: these patients had been treated in the Epilepsiezentrum Kehl-Kork. During a stay of 6–8 weeks their medication therapy was reviewed and changed. In addition, they took part in daily sessions of different kinds of therapy aiming at improving their psychosocial well-being.
RESP: these patients had been treated in the Center of Psychiatry Weissenau, Department of Neurology and Psychiatry with 35 sessions’ respiration feedback according to Fried et al. (1984). The task was to decrease the respiration rate and to increase the end-tidal carbon dioxide (ETCO_2_) by 5%. The aim of this treatment is to avoid hyperventilation, which may elicit seizures.
SCP: these patients had been treated in all study centers (University of Tuebingen, Neurological Clinic Weissenau, and Epilepsiezentrum Kehl-Kork). They received 35 sessions feedback of SCPs. As in the RESP group, the treatment was divided into two phases of 20 and 15 sessions. In addition to the feedback sessions, patients of both groups received daily sessions aiming at transferring the self-regulation skill from the lab into everyday life and improving psychosocial well-being. Patients are instructed to identify seizure prone situations and to use the skill in order to inhibit excessive cortical excitation that might lead into a seizure.

Slow cortical potentials were recorded at Cz, referenced against two electrodes at the mastoids. A session consisted of 145 trials of 8 s duration. Patients were either prompted to produce negative or positive shifts. In less than half of the trials no feedback was given. These transfer trials were introduced to prepare patients to use the skill in their daily life where external feedback is not available.

**Figure 1:**
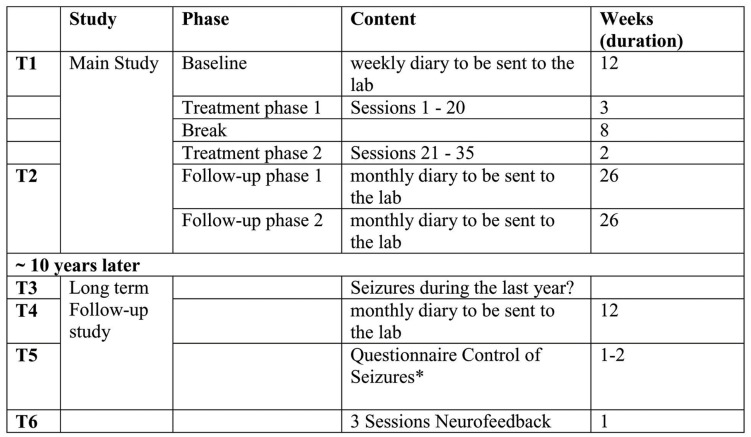
**Timeline of assessments of seizure frequency, treatment, and follow-up evaluations**. *Fragebogen zur Kontrolle von Anfällen (COE, control of epileptic seizures questionnaire) by Trevorrow T and Strehl U, unpublished.

The timeline of the assessments and treatment is depicted in Figure 1.

### Assessment

During the main study seizure frequency, several psychological measures and performance of self-regulation (SCP and RESP groups) were assessed as independent variables. Patients were educated how to fill in the seizure diary by personal as well as by written instructions.

In the long-term follow-up reported here self-regulation of SCP and the assessment of seizure frequency was the main focus. According to the timeline (see Figure 1) seizure frequency was assessed as follows:

T1: baseline before treatment: during the 12 week baseline assessment patients sent the daily diaries weekly by surface mail to their study center.
T2: after the end of treatment: during the 52 weeks follow-up period, patients used the same forms that had to be sent to the centers every month.
T3: patients who wanted to take part in the long-term FU reported the mean seizure frequency per month during the last year. This report was based on whatever the patients had used to recall seizure frequency: specific diaries or marks in their calendars.
T4: 12 weeks baseline similar to the baselines at T1 and T2. Patients had to send their weekly diaries every month to the center (Tuebingen). If they were not sent in time, patients were reminded by phone or e-mail.
T5: as a preparation for the follow-up meeting patients were asked to return a questionnaire about their perceived control over their seizures. One of the questions referred to the number of monthly seizures patients had experienced during the last half-year.
T6: patients took part in three sessions SCP-feedback over two consecutive days. While during the main study a Neurofax amplifier (Nihon Kohden) was used, for this long-term evaluation a special equipment produced for neurofeedback was available (Neuro Prax^®^ from neuroConn GmbH, Germany; http://www.neuroconn.de/startseite_en/) that had been developed according to our requirements. The main difference between the old and new system is in their appearance. The new system is colored and the prompt for the task is a triangle pointing to the top (negativation) or bottom (positivation) of the screen. As cursor for the feedback subjects have the choice between different objects (fish, bird, airplane, boat, and more). The technical details of recording, amplifying, artifact correction, and feedback parameter were comparable to the equipment used in the original study. An additional feature of artifact correction was the eye movement calibration before the start of the recording that allowed online control of artifacts and feedback of these artifacts to the patient. Trials where such an artifact was detected were declared as invalid and were repeated. Each session consisted of 140 trials divided into 5 runs. The succession of runs, their conditions (feedback and transfer) and number of tasks (negativation and positivation) is depicted in Table 3.

**Table 3:** **Composition of tasks and conditions during one session**.

Run number	1	2	3	4	5
Condition	Feedback	Transfer	Feedback	Transfer	Feedback
Trials	40	20	30	20	30
Positivation tasks (%)	60	60	60	60	60

**Figure 2:**
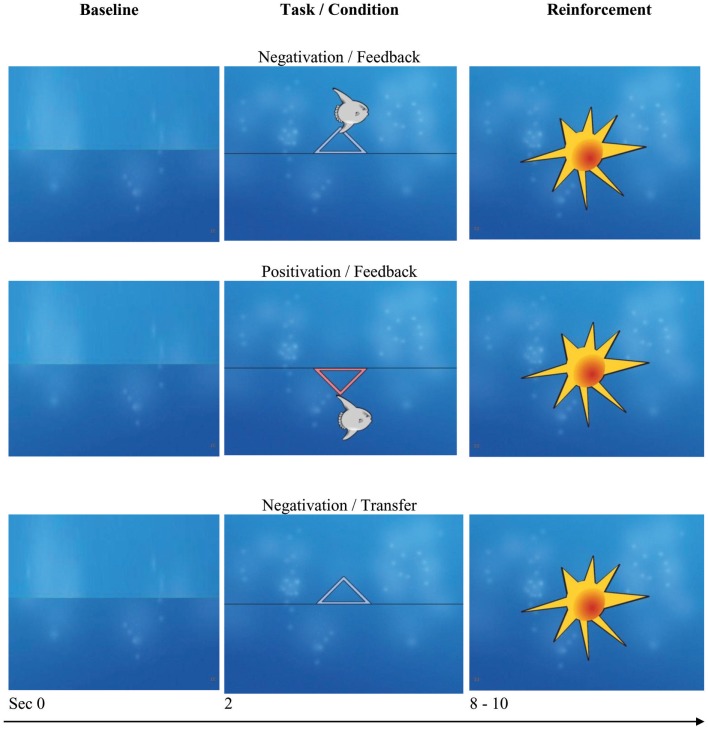
**Discontinuous feedback**. Monitor screens during tasks (negativation and positivation) and conditions (feedback and transfer). If the cursor (in this example a “fish”) is moved in the direction as prompted (up, negativation; down, positivation) a “sun” is shown at the end of the trial. In the case of no success, the screen is empty as shown in the bottom line. Screen shots with friendly permission by neuroConn, Ilmenau, Germany.

The duration of each trial was 8 s. With the appearance of the triangle the trial started and the value taken during the 2 s baseline immediately preceding the trial was set to 0. If the patient succeeded in shifting the potential as required during last third of the trial the picture of a sun appeared on the screen (see Figure 2).

### Data analysis

#### Patients

As depicted in Table 2, three of the 16 patients who took part in the long-term follow-up had undergone surgery due to their epilepsy in the years after the 1 year follow-up. Therefore, their data are not included in the group analysis but if possible will be reported separately.

In order to detect a possible bias due to the self-selection of patients taking part in the long-term follow-up, we compared them with those who refused to participate using the following variables: age, education, age of first seizure, duration of illness, number of AED, number of seizures at T1 and T2, change of seizure rate, pre and post values of IQ. While the variables of seizure frequency were tested with Mann–Whitney *U*-test, all other variables were compared with *t*-tests for independent samples.

#### SCP sessions

Data of all three sessions were analyzed with the “Analyzer” software offered by the Neuro Prax^®^. In preprocessing of data a low pass filter of 4 Hz was used, all trials with eye artifacts as well as trials with amplitudes >200 μV were discarded. For the remaining trials mean values of amplitudes during 5–8 s were computed separately for negativity and positivity tasks during feedback and transfer trials. It was analyzed whether the amplitudes in the positivity tasks and the negativity tasks differed significantly from each other and for both conditions (feedback and transfer). An additional repeated measures analysis of variance (ANOVA) with the factors: Task (negativity/positivity), Session (three sessions), and Condition (feedback/transfer) was conducted. Data were computed only for patients without surgery.

#### Seizures

Because seizure frequencies have a notorious asymmetric distribution, using parametric methods for their analysis requires caution. Therefore, several normalizing transformations were tested, and decimal logarithms proved to yield the best approximation to normality. After this transformation, one patient had to be excluded from the analysis as outlier. A second participant was excluded because his diaries at T4 were not valid. After this, a repeated measures ANOVA with all assessment points was performed with the remaining 11 patients without surgery. In addition, the trends were tested for significance.

Due to the different instruments in assessing seizure frequency all data were analyzed in paired comparison (Wilcoxon-Test, not corrected). Finally, a pre (baseline before treatment) versus post (baseline at long-term evaluation) effect size was computed with Cohen’s *d*.

## Results

### Patients

There was no difference between participants and those patients who were not willing to take part in any of the variables tested.

### Self-regulation of SCP

**Figure 3:**
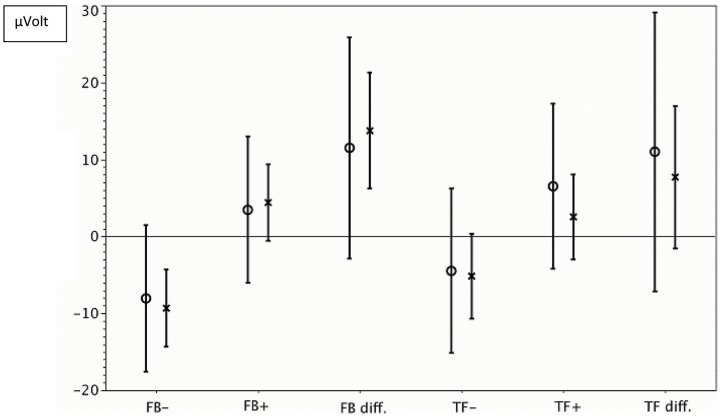
**Self-regulation of SCP**. -x-, mean value and confidence interval for all sessions of patients without surgery; -o-, mean value and confidence interval for all sessions of patients with surgery. FB−, negativity tasks with feedback; FB+, Positivity tasks with feedback. FB diff, difference between shifts during negativity and positivity tasks with feedback. TF+, positivity tasks without feedback (transfer); TF−, negativity tasks without feedback (transfer); TF diff, difference between shifts during negativity and positivity tasks without feedback (transfer).

As indicated by a significant main effect of task [*F*(1,10) = 5.46, *p* = 0.042, η^2^ = 0.35], patients successfully differentiated between the positivity and negativity tasks. The non-significant task by condition interaction (*F* < 1) shows that the correct trend to positive SCP with the positivity task and negative SCP with the negativity task was identical for both feedback and transfer conditions. However, when the data for each condition were analyzed separately, the effect of task was significant only in the feedback condition [*F*(1,10) = 5.73, *p* = 0.038, η^2^ = 0.36], but not in the transfer condition [F(1,10) = 2.48, *p* = 0.15]. Figure 3 depicts the performance in detail for both patients without surgery and with surgery. The interaction between sessions and tasks did not reach significance although for positivity tasks the best results were obtained in session 2. In the negativity task, the amplitudes increased from session to session during feedback but did not reach significance.

### Seizures

The mean seizure frequency at baseline (T1) in the 11 patients who had not undergone surgery was 3.49/week, median 2.46/week. At the different assessment points after treatment, from T2 to T5, the means were between 2.1 and 0.8/week, medians between 1.13 and 0.52/week. As seizures at T1 and T4 were assessed with exactly the same instruments, percentages of seizure decrease were calculated between these two measurement points for each patient, if applicable. Seizure frequency decreased in 8 of 11 patients, in 6 of them the decrease was more than 50%.

One of the 11 patients without surgery who was included in the analysis was seizure free, and 1 of the 3 patients with surgery was seizure free, too.

The ANOVA for all patients without surgery yielded a statistically significant effect of Time with *F*(4,40) = 4.03, *p* = 0.025 (corrected with Greenhouse-Geisser Epsilon). The effect size (η^2^) was 0.287. The linear trend was significant with *F*(1,10) = 8.87, *p* = 0.014, and η^2^ = 0.47.

Pairwise comparisons revealed significant differences (*p* < 0.05) between T1 and each of the following assessment points, with the effect sizes (Cohen’s *d*) between 0.45 (T2–T3 difference) and 0.51 (T1–T4 difference). None of the differences between the later time points (from T3 to T5) attained or even approached significance.

The three patients with surgery had at baseline seizure frequencies of 1.42, 0.91, and 0.54/week, which is below the average frequency for the non-operated patients (3.89/week). In the first of them, the frequency decreased to 0.27/week after surgery, while it did not substantially change in the other two.

## Discussion

Monitoring and follow-up of anti-epileptic treatment is an important issue of the patient–therapist journey (Groenewegen et al., 2014). These authors concluded their multinational patient–physician survey with the statement that successful treatment depends on communication over the long-term. For the group of patients treated with SCPs feedback in the main study (Kotchoubey et al., 2001) who improved although diagnosed as “intractable” the long-term outcome is of special interest. Reduction of seizures after neurofeedback in 74% of patients has been already reported in a meta-analysis (Tan et al., 2009) but long-term data are not available, except the 1 year follow-up of the study reported from our group before (Kotchoubey et al., 2001). A long-term outcome after a period of 9 years after the end of treatment presented in this paper was assessed for the first time. While the original study compared SCPs feedback with two different treatments (review of medication regime and respiratory feedback) only patients of the SCP group were willing to take part in a number sufficient for analysis. One-third of those patients who started the treatment (and 47% of those who completed the 1 year follow-up) again completed seizure diaries and took part in three sessions of neurofeedback. A statistically significant continuous decrease of seizures from the first baseline to the last estimation of seizure frequency was observed. The effect sizes were large for the linear trend and medium for the general linear model. Pairwise comparisons revealed that the decrease did not depend on the assessment instrument (specific diaries to be sent to the lab; regular diaries as usual, estimation in the COE questionnaire). Participants were still able to self-regulate their SCPs as shown by a significant differentiation between negativity and positivity tasks in the feedback condition. Compared with the self-regulation skills during both feedback and transfer demonstrated 6 month after the end of treatment (Kotchoubey et al., 1997) the achievement 10 years later were more moderate, which might partially attributed to the small number of participants. Taking into account the long period without any booster sessions in between it appears surprising that the self-regulation skill was still existent at all. We therefore may assume that participants have successfully acquired a self-regulation skill, which is available for retrieval in their implicit memory. The longest follow-up that showed sustained SCP self-regulation skill reported so far was 2 years in a neurofeedback-study in children with Attention-Deficit-/Hyperactivity-Disorder (Gani et al., 2008).

It would be of interest to analyze a possible correlation between self-regulation performance and reduction of seizures. In an earlier paper with 18 patients at the 6 month follow-up of the main study, it was shown that patients with larger amplitudes of positivity and especially during transfer trials experienced greater improvements (Kotchoubey et al., 1997). A power analysis as regards to the small number of participants in the long-term follow-up revealed that a correlation coefficient of 0.6 would be needed in order to obtain a significant result, which is not realistic to expect.

A comparison with the outcome of other treatments is hardly possible. Long-term follow-up-studies that exceed 3–5 years are rather rare. The longest follow-up we retrieved was 12 years done by Tanriverdi et al. (2008) with patients after temporal lobe surgery (Kunieda et al., 2013), 10 years after resective surgery (Stigsdotter-Broman et al., 2014), and after callosotomy (Ardesch et al., 2007) and 6 years after vagus nerve stimulation (VNS) (Tang et al., 2014). Besides these interventions medication treatments are mostly followed up for a maximum of 3–5 years. For psychobehavioral treatments, long-term results are missing and the sustainability of outcomes are uncertain (Shorvon and Goodridge, 2013). Another difference to our study concerns the method of recording seizure frequency. While we used prospective measures, which were similar before and after treatment, many follow-up studies assessed seizure frequency by phone calls. It is questionable whether this method is adequate, and if yes, whether those results are comparable to ours. Finally, a comparison does not make much sense because the patients in our study had refractory epilepsy, i.e., with no response to medication treatment and not being willing or eligible for surgery. However, a comparison with the results of VNS for medically refractory epilepsy seems to be more justified. Ardesch et al. (2007) report seizure reduction increasing from 14% after the first year to 50% after 6 years follow-up in a group of 19 patients (Tang et al., 2014). Contrary to other studies (e.g., Sperner et al., 2005), which state that the early response predicts the long-term outcome, in the VNS study, the improvements were small at the beginning, increasing in the following years (Tang et al., 2014). A similar development was observed in our study, but percentages increased from 40 to 76%. According to a pilot study (Brodie, 2013) both VNS and SCP treatment might have a common mechanism of effect, namely, the increase of excitation thresholds of cortical neurons. An advantage of SCP-feedback compared to VNS is not only the bigger decrease of seizures but also the absence of side effects. Taking these results into consideration, it may be suggested that SCP-feedback might be recommended as a novel approach, which may meet the demands in treating patients with refractory epilepsies (Hoppe et al., 2007).

Of course several limitations have to be acknowledged. First of all, we were not able to compare with the control groups of the original study as not enough patients from these groups were willing to take part. One reason why only 17% (MED) and 18% (RESP) patients responded could be that these patients had been contacted before only by their study centers but not by the University of Tuebingen, now asking for their participation. Due to constraints set up by the Ethics Committee, we were not allowed to ask for reasons for no participation. Therefore, we cannot exactly estimate the impact of these missing data on the results but it is worth noticing that we did not find any difference in demographic and seizure characteristic between those who took part and those who did not. Another limitation may concern the reliability of the number of seizures as indicated by the patients. Several authors who tried to obtain greater accuracy by ambulatory or in-patient EEG assessment report that around 50% of seizures are not declared (e.g., Kerling et al., 2006; Tanriverdi et al., 2008). This problem is not unique to our study. Instead, it has to be taken into account by any study (and any report in the doctor’s office) that relies on patients’ statements. It is all the more important that we used identical forms to record seizures in three of five assessment points. Finally, the impact of confounding variables throughout all the years that passed by since the end of the treatment has to be considered (which is also true for other studies). Due to the small number of patients we were not able to assess these variables on a systematic basis. We do not know what impact possible positive changes in the quality of life, education, occupation, or family status might have had on the seizures. Neither do we know what impact the reduction of seizures can have on the aforementioned variables. An exact control of the effect of everyday life would require a huge number of patients at the beginning of a study. If this would be a condition *sine qua non-the* majority of long-term studies done so far are done in vain.

To summarize the main limitation of this study is the small number of patients that do not allow us to draw causal conclusions. Notwithstanding this small sample size, statistically significant long-term reduction of seizures and sustained self-regulation of SCPs were observed. It has to be noted that this success was achieved without booster sessions. Future research should not only try to replicate these promising results but also include SCP-feedback treatment as an option in the treatment of patients with (intractable) epilepsies.

## Conflict of Interest Statement

The authors declare that the research was conducted in the absence of any commercial or financial relationships that could be construed as a potential conflict of interest.
